# Recurrence Patterns After Resection of Sacral Chordoma: Toward an Optimized Postoperative Target Volume Definition

**DOI:** 10.3390/cancers17152521

**Published:** 2025-07-30

**Authors:** Hanna Waldsperger, Burkhard Lehner, Andreas Geisbuesch, Felix Jotzo, Eva Meixner, Laila König, Sebastian Regnery, Katharina Kozyra, Lars Wessel, Sandro Krieg, Klaus Herfarth, Jürgen Debus, Katharina Seidensaal

**Affiliations:** 1 Department of Radiation Oncology, Heidelberg University Hospital, 69120 Heidelberg, Germany; hanna.waldsperger@med.uni-heidelberg.de (H.W.);; 2 Heidelberg Institute of Radiation Oncology (HIRO), 69120 Heidelberg, Germany; 3 National Center for Tumor Diseases (NCT), 69120 Heidelberg, Germany; 4 Heidelberg Ion-Beam Therapy Center (HIT), Department of Radiation Oncology, Heidelberg University Hospital, 69120 Heidelberg, Germany; 5 Center for Orthopedics, Trauma Surgery and Paraplegiology, University of Heidelberg, 69118 Heidelberg, Germany; 6 Department of Neurosurgery, Heidelberg University Hospital, 69120 Heidelberg, Germany; 7 German Cancer Consortium (DKTK), Partner Site Heidelberg, 69120 Heidelberg, Germany; 8 Clinical Cooperation Unit Radiation Oncology, German Cancer Research Center (DKFZ), 69120 Heidelberg, Germany

**Keywords:** sacrococcygeal chordoma, protons, carbon ions, protons, radiotherapy, recurrence patterns, sacrectomy

## Abstract

**Background:** Postoperative recurrence of sacrococcygeal chordomas presents significant clinical challenges due to unusual recurrence patterns. This study aimed to characterize these patterns of recurrence to inform improved adjuvant radiotherapy planning. **Methods:** We retrospectively analyzed 31 patients with recurrent sacrococcygeal chordoma following surgery, assessing recurrence locations considering initial tumor extent, resection levels, and postoperative anatomical changes on MRI. In 18 patients, pre- and postoperative imaging enabled the spatial mapping of early recurrence origins relative to the initial tumor volume using isotropic expansions. The median initial gross tumor volume was 113 mL. **Results:** Recurrences were mostly multifocal and predominantly involved soft tissues (e.g., mesorectal/perirectal space (80.6%), piriformis and gluteal muscles (80.6% and 67.7%, respectively) and osseous structures, particularly the sacrum (87.1%)). The median time to recurrence was 15 months. The initial surgery was R0 in 17 patients (55%). The highest infiltrated sacral vertebra was S1 in 3%, S2 in 10%, S3 in 35%, S4 in 23%, S5 in 10%, and coccygeal in 19%. Anatomical changes post-resection, including rectal herniation into gluteal and subcutaneous tissues, significantly affected radiotherapy planning. Expansion of the initial tumor volume by 2 cm failed to encompass all recurrence origins in 72% of cases. A 5 cm expansion was required to achieve full coverage in 56% of patients, though 22% of recurrences still lay beyond this margin and the remaining were covered only partially. **Conclusions:** Recurrent sacrococcygeal chordomas exhibit complex, soft-tissue-dominant patterns and are influenced by significant anatomical displacement post-surgery. Standard target volume expansions are often insufficient to cover the predominantly multifocal recurrences.

## Simple Summary

Sacrococcygeal chordomas present a rare malignancy that are challenging to treat due to their locally aggressive nature. The main treatments are surgical resection and radiotherapy. Wide en bloc surgery aims to reduce recurrence but is often limited by anatomical constraints, especially with large tumors or those involving critical neurovascular structures. This study retrospectively analyzed 31 patients with recurrent sacrococcygeal chordoma after surgery, focusing on recurrence patterns and locations. Results showed that recurrences were mostly multifocal, involving soft tissues like the mesorectal space, gluteal muscles, and the sacrum. The median time to recurrence was 15 months. Postoperative anatomical changes, such as rectal herniation, complicated radiotherapy planning. Expanding the initial tumor volume by 2 cm often failed to cover all recurrences, with a 5 cm margin needed in many cases, yet some recurrences still occurred outside this range. These findings highlight the complex, multifocal nature of recurrences, and the need for individualized radiotherapy planning.

## 1. Introduction

Sacral chordoma is a rare and malignant bone tumor that poses a significant therapeutic challenge due to its locally aggressive behavior [1,2]. These tumors arise from residual notochordal cells and can develop in the sacrococcygeal region, as well as in the skull base and mobile spine. Despite their slow-growing nature, sacral chordomas have a high tendency for local recurrence and late metastatic spread. Their close proximity to critical structures makes complete surgical removal difficult and contributes to the complexity of disease management.

Surgical resection and definitive particle therapy represent the two main treatment modalities for sacral chordomas. While wide en bloc surgical excision is considered one standard approach to reduce the risk of local recurrence, it is frequently limited by anatomical constraints, particularly when tumors are large or involve critical neurovascular structures [3,4]. Consequently, wide resections are achieved in only 40–55% of cases [5,6,7,8]. In cases where surgery results in marginal or intralesional resections, the risk of local recurrence increases significantly. Moreover, a substantial number of patients present with tumors that are not amenable to complete surgical resection, making definitive particle therapy a critical alternative [7,9,10,11]. Postoperative radiotherapy has been commonly employed to enhance local control, especially in cases with positive or close surgical margins [12]. The highest rates of local control and survival were previously described with early adjuvant radiation therapy [13], while local control rates of salvage radiotherapy were significantly lower (5-year LC of 88% vs. 9%, respectively) [14]. High-dose radiation therapy with doses above 70–77 Gy [15], particularly with protons or carbon ions, has demonstrated efficacy in reducing recurrence rates [16]. However, despite its widespread application, the optimal delineation of the postoperative target volume remains unclear. Currently, no standardized guidelines exist to define the precise clinical target volume (CTV), leading to variations in treatment.

To address this gap, we analyzed recurrence patterns in patients who undergo surgical resection without adjuvant radiotherapy. Understanding the anatomical distribution of local recurrences in these cases can provide critical insights into the regions at highest risk of tumor regrowth. This information could serve as a foundation for establishing evidence-based guidelines for postoperative target volume delineation, optimizing radiotherapy planning. In this study, we aim to systematically evaluate recurrence patterns following surgery alone to refine postoperative target volume definitions and enhance treatment strategies for sacral chordoma.

## 2. Methods

This retrospective study was conducted at the Heidelberg University Hospital and the Heidelberg Ion-Beam Therapy Center (HIT). Ethical approval was obtained from the Heidelberg University ethics committee (S-488/2024). We screened all patients with sacroccocygeal chordoma who received carbon ions or protons at the HIT from 2012 until 2025 and identified 31 out of 214 patients who presented with local recurrence following surgery alone. Patient selection criteria included diagnosis of chordoma recurrence, prior surgical treatment history, and suitability for salvage particle therapy. Patients after incomplete resection (R2) were excluded. Data were collected through a retrospective analysis of medical records. The median time from first resection to recurrence was measured from first resection until the appearance of recurrence on MRI. The extension of local recurrence was analyzed on the planning MRI or the MRI before re-resection. All patients received a CT scan as part of the radiation treatment planning. The initial and recurrence imaging were registered to the planning CT scan. The initial tumor volumes (GTVinit) were isotropically expanded by a 2 cm, 3 cm, and 5 cm margin to analyze the possible coverage of the early recurrence origins. The recurrence origins were defined as the expansion of the recurrence lesions on their first appearance on MRI. Two out of 31 patients presented with re-recurrence after resection of the first recurrence (Patient nr. 9 and 21) and were analyzed for the recurrence and re-recurrence pattern. The local control and survival data for patients treated following incomplete resections or for local recurrences will be published in a separate study. Ten patients were treated within the phase II study ISAC, the results were published previously by our group [17]. Analyses were performed using SPSS v29.

## 3. Results

### 3.1. Patient Characteristics

A total of 31 patients were included in the analysis. Of these, 18 (58%) were male and 13 (42%) were female. The median age at first diagnosis was 60 years (range: 26–78 years). Brachyury staining was positive in 11 (36%) patients and missing in 20 (64%) cases. At the time of first diagnosis, the highest infiltrated sacrococcygeal vertebra was identified as follows: S1 in 1 (3%), S2 in 3 (10%), S3 in 11 (35%), S4 in 7 (23%), S5 in 3 (10%), and coccygeal in 6 (19%) patients. Regarding the pathological resection status of the first surgery, 7 (22%) had R0 resection, 10 (32%) had R0 (close), 8 (26%) had R1, 3 (10%) had RX (fragmented), and data were missing in 3 (10%) patients.

Patients with RX status (i.e., fragmented pathological specimen) or missing pathological R-status were evaluated in detail based on surgical reports, pathology documentation, and imaging. One patient with RX status was included in the recurrence origin analysis due to intraoperatively assessed complete resection. Another patient with missing pathological R-status was also included, as the surgical report indicated intraoperatively assessed complete resection, and immediate postoperative imaging showed no evidence of residual disease. In contrast, two patients—one with RX status and one with missing R-status—were excluded from the recurrence analysis due to lack of preoperative imaging. Two additional patients with missing R-status were excluded due to insufficient documentation in both surgical and pathological records.

As the Enneking classification was not used even in one of the surgical reports, we decided not to use and reassess it retrospectively. The median time from first resection to first recurrence was 15 months (range: 2–68 months), and the median time from first diagnosis to first recurrence was 17 months (range: 2–70 months). In 65% of the patients the recurrence occurred within 2 years after resection and in 48% of the patients within 12 months. The median time from first diagnosis to first presentation at the HIT for particle therapy was 30 months (range: 9–167 months); several patients (N = 11, 35%) had a further resection of the recurrence. Radiation therapy was then performed at the second recurrence or after incomplete resection. MRI findings at recurrence showed a unifocal lesion in 3 (10%) of the patients and multifocal lesions in 28 (90%) (Table 1).

### 3.2. Recurrence Patterns

**Figure 1:**
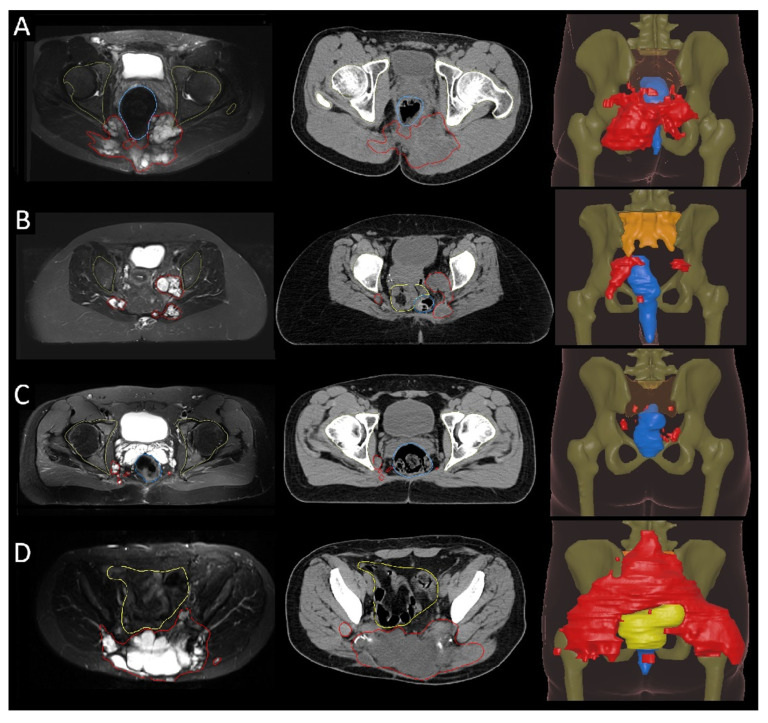
Examples of sacrococcygeal chordoma recurrence in four patients following sacrectomy. Representative axial and sagittal slices from MRI and CT are shown alongside corresponding 3D models. All cases (**A**–**D**) demonstrate multifocal soft-tissue recurrence (red); note that residual sacrum (orange) is displayed as transparent in (**B**,**D**) for better visualization. Other depicted structures are the rectum (blue), the acetabulum, and femoral heads (olive green), and the bowel (bright yellow).

**Figure 2:**
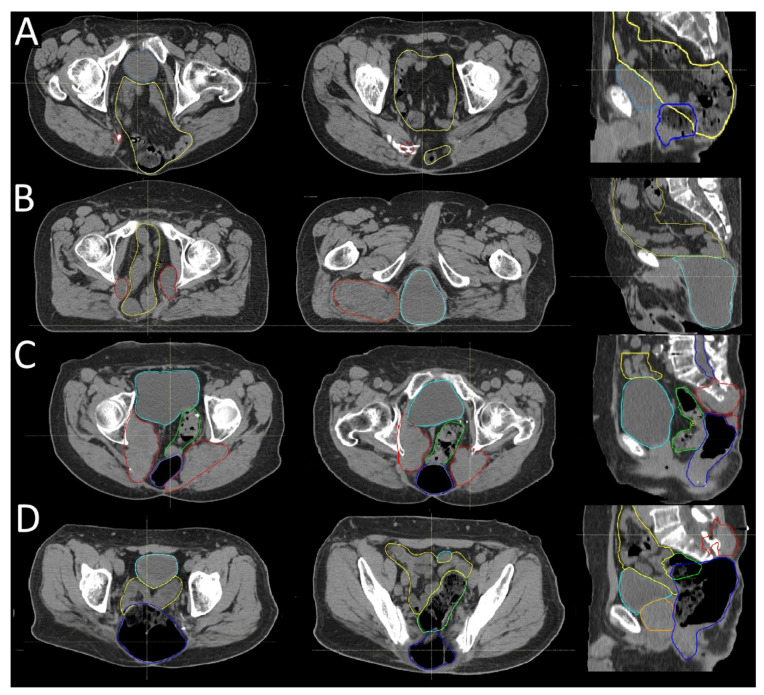
Anatomical changes following sacrectomy. Representative axial and sagittal CT slices from four sacral chordoma patients after surgery. In image (**A**), the small bowel (yellow) is visible. In image (**B**), the rectum has been surgically removed, and the bladder (light blue) now occupies that anatomical region. Images (**C**,**D**) show a typically distended rectum (dark blue) located close to the skin surface. Recurrent chordoma lesions are marked in red. Other depicted structures are the prostate (orange) and sigmoid collon (green).

**Figure 3:**
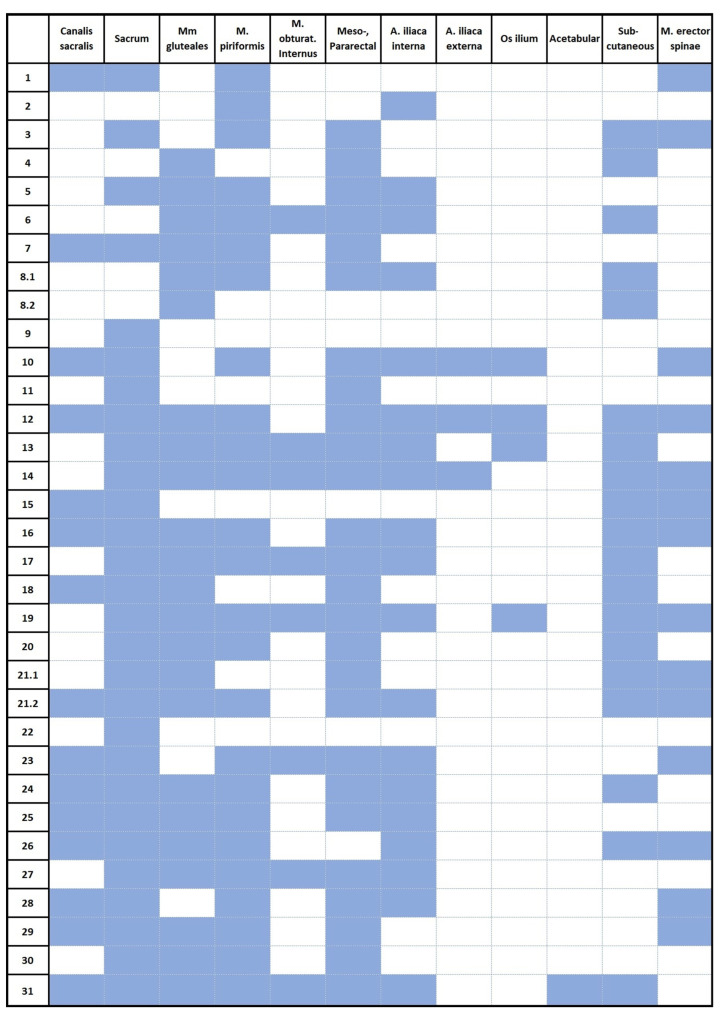
Recurrence patterns. Patient numbers and recurrence localizations. Patient numbers 8.1 and 8.2 and 21.1 and 21.2 were analyzed for initial recurrence as well as re-recurrence after resection of the first recurrence.

Recurrences predominantly involved the sacrum and soft tissue structures (Figure 1, Figure 2 and Figure 3). Recurrences in osseous structures most frequently involved the canalis sacralis (15 patients, 48.4%) and the sacrum, occurring both along the osseous resection margin and in regions of the sacrum beyond the resection site (27 patients, 87.1%). Notably, these distant sacral recurrences tended to arise near the resection area rather than in remote sacral vertebrae. In many cases, recurrences that originated at the resection site extended into adjacent sacral segments, making it challenging to differentiate small local recurrences in vertebrae above the resection level. As a result, recurrences at and near the resection site were collectively analyzed as a single sacral recurrence region. Soft tissue extension involved several pelvic and perirectal regions, including the gluteal musculature (21 patients, 67.7%), musculus piriformis (25 patients, 80.6%), obturator region (8 patients, 25.8%), and mesorectal/perirectal tissue (25 patients, 80.6%). The internal (19 patients, 61.3%) and external iliac regions (3 patients, 9.7%) were also frequently affected. Osseous involvement beyond the sacrum included the os ilium (4 patients, 12.9%) and acetabulum (1 patient, 3.2%). Infiltration of the subcutaneous tissue was seen in 17 patients (54.8%), and the autochthonous back muscles were involved in 13 patients (41.9%) (Table 2 and Figure 1). Overall, the recurrence pattern showed a tendency for locally extensive, multifocal soft tissue infiltration, often spreading into adjacent muscular and pelvic compartments.

### 3.3. Postoperative Radiotherapy Planning Based on Recurrence Patterns

In a subset of 18 patients with available MRI scans of both the initial tumor and early recurrences—representing the likely site of recurrence origin—an analysis was performed to determine whether standard isotropic margin expansions would have sufficiently covered the recurrence origins. The initial tumor volumes (GTVinit) were isotropically expanded by 2 cm, 3 cm, and 5 cm. The median initial gross tumor volume was 113 mL (range: 31–419 mL) (Table 1). With a 2 cm margin the recurrences were partially included in 28% of the patients, and in 72% we found isolated recurrence origins outside the expanded volume. With a 3 cm margin the recurrences were fully covered in 11% of the patients and partially covered in 44.5% of the patients. In 44.5% of the patients not all of the recurrence origins were covered. An expansion of 5 cm encompassed all recurrence origins in 56% of the patients, showed partial coverage in 22%, and recurrences outside the margins in 22% of the patients (Table 3).

In a subset of 14 patients, we observed herniation of the rectum through the gluteal musculature, with displacement extending subcutaneously below the skin surface (Figure 2). These patients had predominantly undergone resection at the level of S2 (21%) and S3 (50%). In one patient who had undergone resection of the rectum, a herniation of the bladder through the gluteal muscles was also noted. This anatomical alteration significantly influenced the postoperative dose distribution, as the displaced organs shifted into areas not typically included in standard target volumes, posing challenges for both dose coverage and organ-at-risk sparing (Figure 2).

## 4. Discussion

To our knowledge, this is the first study to comprehensively analyze local recurrence patterns following sacrectomy. In our analysis, we observed that recurrences following sacrectomy for sacral chordoma were predominantly multilocular, with disease reappearing in multiple compartments of the pelvis. Notably, these multifocal recurrences were frequently located in the soft tissues, particularly within the piriformis and gluteal muscles, as well as within the small pelvis, e.g., lateral to the rectum. This pattern suggests a tendency for tumor spread beyond the immediate surgical bed, which may not always be adequately covered in standard postoperative radiation target volumes. The propensity for multifocal recurrence highlights the challenge of achieving durable local control.

En bloc R0 resection has been described as the most important prognostic factor for local control and thus overall survival. Achieving negative margins is essential in reducing the risk of recurrence, further emphasizing the importance of careful patient selection [7]. Identifying patients who are optimal surgical candidates—based on tumor extent, anatomic feasibility of resection, and likelihood of achieving an R0 margin—is critical. The morbidity risk of sacrectomy is highly dependent on the level of tumor infiltration. While resections in the lower sacral region (S4–S5) are often associated with fewer functional impairments, high-level infiltrations at S1–S3 carry a significantly increased risk of neurological deficits, impaired mobility, and bowel, bladder, and sexual dysfunction [18,19,20,21,22]. In a publication of 27 cases from the University Hospital Dresden, sacrectomy was associated with a high complication rate, with 81.5% of patients experiencing at least one minor-to-moderate complication, predominantly wound-healing disorders and infections [23]. These potential complications must be carefully weighed against the expected oncologic benefit, particularly in cases where achieving an R0 resection may be challenging. For patients in whom an R0 resection is unlikely, alternative treatment strategies should be considered, especially given our observation that multilocular recurrences can develop within one to two years after surgery. Similarly, in a publication of 18 chordoma cases, local recurrence occurred in 12 patients (66%) at a mean of 29 months postoperatively; among the 10 patients with wide surgical margins, 60% experienced local recurrence [24], while other authors report local recurrence rates of 30% after wide resection and 63–67% after marginal or intralesional resection [6]. In this context, particle therapy with carbon ions or protons has emerged as a promising option, offering high-dose, conformal radiation with superior normal tissue sparing. Several studies have demonstrated favorable local control rates making it a viable alternative or adjunct to surgery in select cases [25,26,27,28,29]. In a multicenter study involving 911 patients, Yolcu et al. examined the effectiveness of carbon ion radiotherapy (CIRT) in chordoma patients deemed non-resectable. The findings revealed no significant difference in five-year overall survival, local recurrence-free survival, or metastasis-free survival when compared to patients who underwent margin-free en bloc resection but significantly lower rates in motor neuropathy. Their propensity-matched analysis showed that margin-positive surgery without adjuvant radiotherapy (median OS: 60.6 months) was associated with lower overall survival (OS) compared to CIRT (median OS: 64.7 months, *p* = 0.03). Additionally, CIRT demonstrated significantly better OS compared to primary radiotherapy alone (median OS: 64.9 vs. 31.8 months, *p* < 0.001), reinforcing its potential role in improving local control [30].

Postoperative anatomical changes following sacrectomy often result in the rectum being positioned dorsally, just beneath the skin. This altered anatomy can pose challenges for the effective irradiation of multifocal recurrences, even when utilizing advanced modalities like proton or carbon ion therapy. The proximity of the rectum to the skin increases the risk of radiation-induced toxicity, limiting the deliverable dose to recurrent tumor sites.

Local recurrence after definitive radiotherapy with carbon ions typically manifests as regrowth of the treated tumor following an initial reduction in size, which can occur after several years. With clinical target volume (CTV) margins of 2 cm and planning target volume (PTV) margins of 5–7 mm, the risk of locoregional metastases is exceptionally low [17]. In contrast, we observed several cases of multifocal recurrence within the small pelvis after sacrectomy even in a short time-span after en bloc resection, with recurrences occurring not only within a 2 or 3 cm margin around the initial tumor but also at sites considerably distant from the original location. This pattern may result from tumor cell dissemination during surgery, particularly if tumor rupture and spillage of the gelatinous mass occur, which are known risk factors for local recurrence and locoregional spread. Furthermore, surgical manipulation itself may act as a trigger for recurrence with a surprisingly fast regrowth as observed in this cohort. In our study, all patients except three were referred from external institutions, limiting our access to operative reports to confirm whether such intraoperative events contributed to the multifocal recurrence observed. Notably, the median initial gross tumor volume (GTV) in this study was 113 mL, which is smaller than the median GTV of 185 mL reported in our trial of definitive hypofractionated radiation therapy for sacrococcygeal chordoma.

The definition of the postoperative radiation target volume plays a crucial role in ensuring adequate local control while minimizing unnecessary radiation exposure. Previous recommendations often suggested including the entire sacrum and coccyx to account for potential microscopic disease and reduce recurrence risk [12]. However, our findings indicate that recurrences predominantly occur in the soft tissues, at the caudal sacrum, and in additional intrapelvic locations such as the perirectal space, rather than diffusely within the residual sacral bone. This suggests that extensive sacral coverage may not always be necessary. Based on our findings and previous experience from definitive radiotherapy [17], we recommend applying a 2 cm margin from the preoperative gross tumor volume (into the residual sacrum, extending up to a maximum of two cranial sacral vertebrae, to ensure the adequate coverage of potential microscopic disease while minimizing unnecessary radiation to uninvolved bone. Particularly in soft tissues, our findings suggest that standard isotropic expansions may fail to adequately cover anatomical regions at risk. Based on the observed recurrence patterns, we propose that future CTV definitions should more precisely account for the perirectal space, the gluteal, and piriformis muscle regions. Where technically feasible, the margin into the soft tissues should be extended to 3–5 cm, including all postoperative changes visible on MRI—particularly in cases of incomplete resection or suspected tumor cell dissemination. While this approach requires prospective validation, it may enable the improved coverage of subclinical disease. Given the high relapse rates and limited prognosis associated with recurrent chordoma, incorporating tailored target volume strategies during initial therapy is especially important [10]. Adjuvant radiotherapy for sacral chordomas requires high doses, exceeding 70 Gy EQD2, to achieve effective local control. However, based on our clinical experience with anatomical changes following sacrectomy, achieving adequate margin coverage in the constrained anatomy of the small pelvis—particularly at dose levels exceeding 70 Gy—remains challenging, even with advanced techniques such as proton or carbon ion therapy, and encompassing significant portions of the pelvic organs within the target volume is unfeasible without risking substantial radiation-induced toxicity. Therefore, meticulous surgical techniques are essential to prevent tumor dissemination. This underscores the critical importance of careful patient selection and multidisciplinary planning prior to surgical intervention.

Candidates for surgery should be those in whom a margin-negative (R0) resection is highly likely, as this offers the best chance of disease control. In contrast, margin-positive (R1) or intralesional (R2) resections should be strictly avoided, as they may not only fail to provide a therapeutic benefit but could also contribute to tumor cell dissemination and multifocal recurrence. In cases where an R0 resection is unlikely, definitive radiotherapy should be considered as an alternative treatment approach.

Limitations of our dataset include its retrospective design and the lack of standardized surgical documentation. Given the large number of referring institutions and the variability in surgical practice, consistent use of such classifications remains challenging. Nonetheless, we recommend routine documentation in future clinical practice to facilitate multidisciplinary treatment planning and improve the quality of outcome reporting in such a rare condition as chordoma. Additionally, the uniform use of the Enneking classification in the surgical reports is recommended. Notably, Brachyury immunostaining was not routinely performed a decade ago and was therefore unavailable for the majority of patients. Less complex or more straightforward recurrences might have been managed elsewhere and not referred to our center, potentially skewing the case mix in favor of more challenging presentations.

## 5. Conclusions

Our analysis of local recurrence patterns following sacrectomy for sacral chordoma reveals a predominant occurrence of multifocal recurrences across various pelvic compartments, including the piriformis and gluteal muscles, as well as intrapelvic regions such as the perirectal space. This distribution suggests a propensity for tumor spread into adjacent musculature and perirectal areas, often extending beyond the immediate surgical bed, which may not be sufficiently encompassed by standard postoperative radiation target volumes.

In conclusion, our study contributes significantly to the understanding of recurrence patterns in sacral chordoma and underscores the importance of individualized treatment strategies. A multidisciplinary approach, integrating meticulous surgical techniques with advanced radiotherapy planning, is paramount to improving patient outcomes in this challenging malignancy. Further prospective studies to validate our findings in postoperative target volume delineation are essential.

## Figures and Tables

**Table 1:** Patient characteristics.

N	31
Sex (N, %)	
Male	18 (58)
Female	13 (42)
Age at first diagnosis (years)	
Median (Range)	60 (26–78)
Brachyury-staining (N, %)	
Positive	11 (36)
Negative	0
Missing	20 (64)
Highest infiltrated sacrococcygeal vertebra at first diagnosis (N, %)	
S1	1 (3)
S2	3 (10)
S3	11 (35)
S4	7 (23)
S5	3 (10)
Coccygeal	6 (19)
Volume of initial tumor (gross tumor volume, GTV, in mL) (Median, range)	
Median (Range)	113 (31–419)
Missing (N, %)	10 (32)
Pathological resection status of the first resection (N, %)	
R0	7 (22)
R0 (close)	10 (32)
R1	8 (26)
RX (fragmented)	3 (10)
Missing	3 (10)
Time from first resection to first recurrence (months)	
Median (Range)	15 (2–68)
Time from first diagnosis to first recurrence (months)	
Median (Range)	17 (2–70)
Time from first diagnosis to first presentation at the HIT (months)	
Median (Range)	30 (9–167)
Resection of recurrence before irradiation (N, %)	
Yes	11 (36)
No	20 (64)
Extension on recurrence on MRI (N, %)	
Unifocal lesion	3 (10)
Multifocal lesions	28 (90)

**Table 2:** Recurrence pattern.

Total (N)	31	
Localization	Recurrence (N)	Percentage
Sacrum	27	87.1%
Meso-, Pararectal	25	80.6%
M. piriformis	25	80.6%
Mm. gluteales	21	67.7%
A. Iliaca interna	19	61.3%
Subcutaneous	17	54.8%
Canalis sacralis	15	48.4%
M. erector spinae	13	41.9%
M. obturatorius internus	8	25.8%
Os ilium	4	12.9%
A. Iliaca externa	3	9.7%
Acetabular	1	3.2%

Sacrum includes the osseous resection margin and sacral lesions distant from the resection site, A. iliaca interna includes the iliacal intern region and the region around the branches of the artery.

**Table 3:** Coverage of recurrence origins by the expansion of initial gross tumor volume (GTVinit).

N = 18			
Expansion	Full Coverage of All Recurrence Origins (N, %)	At Least Partial Coverage of All Recurrence Origins (N, %)	At Least One Recurrence Origin Outside the Margin (N, %)
GTVinit + 2 cm	0 (0)	5 (28)	13 (72)
GTVinit + 3 cm	2 (11)	8 (44.5)	8 (44.5)
GTVinit + 5 cm	10 (56)	4 (22)	4 (22)

Subset of 18 patients with available MRI scans of both the initial tumor and early recurrences.

## Data Availability

The data can be shared upon request.
